# Understanding the Needs and Lived Experiences of Patients With Graft-Versus-Host Disease: Real-World European Public Social Media Listening Study

**DOI:** 10.2196/42905

**Published:** 2023-11-10

**Authors:** Zinaida Perić, Grzegorz Basak, Christian Koenecke, Ivan Moiseev, Jyoti Chauhan, Sathyaraj Asaithambi, Alexandros Sagkriotis, Sibel Gunes, Olaf Penack

**Affiliations:** 1 School of Medicine University Hospital Center Zagreb University of Zagreb Zagreb Croatia; 2 Department of Hematology Transplantation and Internal Medicine Medical University of Warsaw Warsaw Poland; 3 Hannover Medical School Hannover Germany; 4 RM Gorbacheva Research Institute Pavlov University St. Petersburg Russian Federation; 5 Novartis Healthcare Pvt Ltd Hyderabad India; 6 Novartis Pharmaceuticals AG, Basel Basel Switzerland; 7 Gilead Sciences Europe Ltd Uxbridge United Kingdom; 8 Department of Hematology, Oncology, and Tumor Immunology Charité – Universitätsmedizin Berlin, corporate member of Freie Universität Berlin and Humboldt-Universität zu Berlin Berlin Germany

**Keywords:** graft-versus-host disease, GVHD, infoveillance, patient journey, quality of life, real-world evidence, social media listening, social media

## Abstract

**Background:**

Graft-versus-host disease (GVHD) is the major cause of short- and long-term morbidity and mortality after allogeneic hematopoietic stem cell transplantation. Treatment options beyond corticosteroid therapy remain limited, and prolonged treatment often leads to impaired quality of life (QoL). A better understanding of the needs and experiences of patients with GVHD is required to improve patient care.

**Objective:**

The aim of this study is to explore different social media (SM) channels for gathering and analyzing the needs and experiences of patients and other stakeholders across 14 European countries.

**Methods:**

We conducted a retrospective analysis of SM data from the public domain. The Talkwalker social analytics tool collected data from open-access forums, blogs, and various social networking sites using predefined search strings. The raw data set derived from the aggregator tool was automatically screened for the relevancy of posts, generating the curated data set that was manually reviewed to identify posts that fell within the predefined inclusion and exclusion criteria. This final data set was then used for the deep-dive analysis.

**Results:**

A total of 9016 posts relating to GVHD were identified between April 2019 and April 2021. Deduplication and relevancy checks resulted in 325 insightful posts, with Twitter contributing 250 (77%) posts; blogs, 49 (15%) posts; forums, 13 (4%) posts; Facebook, 7 (2%) posts; and Instagram and YouTube, 4 (1%) posts. Patients with GVHD were the primary stakeholders, contributing 63% of all SM posts. In 234 posts, treatment was the most discussed stage of the patient journey (68%), followed by symptoms (33%), and diagnosis and tests (21%). Among treatment-related posts (n=159), steroid therapy was most frequently reported (54/159, 34%). Posts relating to treatment features (n=110) identified efficacy (45/110, 41%), side effects (38/110, 35%), and frequency and dosage (32/110, 29%), as the most frequently discussed features. Symptoms associated with GVHD were described in 24% (77/325) of posts, including skin-related conditions (49/77, 64%), dry eyes or vision change (13/77, 17%), pain and cramps (16/77, 21%), and fatigue or muscle weakness (12/77, 16%). The impacts of GVHD on QoL were discussed in 51% (165/325) of all posts, with the emotional, physical and functional, social, and financial impacts mentioned in 69% (114/165), 50% (82/165), 5% (8/165), and 2% (3/165) of these posts, respectively. Unmet needs were reported by patients or caregivers in 24% (77/325) of analyzed conversations, with treatment-related side effects being the most common (35/77, 45%) among these posts.

**Conclusions:**

SM listening is a useful tool to identify medical needs. Treatment of GVHD, including treatment-related side effects, as well as its emotional and physical impact on QoL, are the major topics that GVHD stakeholders mention on SM. We encourage a structured discussion of these topics in interactions between health care providers and patients with GVHD.

**Trial Registration:**

Not applicable

## Introduction

Graft-versus-host disease (GVHD) is a systemic immune-related complication of allogeneic hematopoietic stem cell transplantation (HSCT) and is a major cause of short- and long-term morbidity and no relapse mortality [1,2]. GVHD occurs in two main forms: acute GVHD (aGVHD) and chronic GVHD (cGVHD), each of which is defined by distinct clinical presentations [3,4].

Treatment of GVHD remains challenging. Corticosteroids are the standard first-line therapy for both aGVHD and cGVHD, with response rates ranging from 40% to 60%, which highlights an urgent unmet need for the steroid-refractory patient population [5]. Several interventions, including extracorporeal photopheresis, Janus kinase (JAK) inhibitors, and other immunosuppressive therapies, are used for second-line therapy, although efficacy data for these interventions are limited [6-9]. Over the past 5 years, the US Food and Drug Administration has granted 4 approvals to therapies for the treatment of GVHD [10]. Ruxolitinib, a small-molecule JAK1/2 inhibitor, has received approval for the treatment of adult and pediatric patients with either steroid-refractory aGVHD or steroid-refractory and steroid-dependent cGVHD after failure of 1 or 2 lines of systemic therapy. Ibrutinib, a potent small-molecule Bruton’s tyrosine kinase inhibitor, was approved for adult patients with cGVHD after failure of 1 or 2 lines of systemic therapy. In addition, belumosudil, an oral selective Rho-associated kinase 2 inhibitor, has been approved for adult and pediatric patients with cGVHD after failure of at least 2 previous lines of systemic therapy [10].

Impaired quality of life (QoL) is often reported in patients with GVHD, particularly in those with cGVHD who experience physical challenges. In addition to reduced QoL, cGVHD has been associated with low functional status and high symptom burden [11-14]. A patient-reported outcomes study with patients who have ongoing cGVHD highlighted that 26.7%-39.4% of patients were unable to work due to health-related issues, compared with 12.1% whose cGVHD had resolved and 15.4% who did not have cGVHD [13]. Patients with moderate or severe cGVHD were more likely to take prescription drugs for pain, anxiety, and depression when compared with those who had resolution of GVHD [13]. The emotional impact of cGVHD was noted in a study of patients from the Chronic GVHD Consortium (N=482), with approximately one-fifth of patients having clinically significant depression or anxiety, of which depression was associated with lower overall survival [15]. A further prospective study (N=52) identified approximately one-third of patients with clinically significant depression or anxiety [16].

Social media (SM) has been widely used for health-related purposes, including health campaigns, medical education, and disease surveillance [17]. Patients can use SM for diverse reasons, including increasing disease knowledge, expression of emotions, experience-sharing of their disease and treatments, contact and community, and advice-gathering [18]. The data generated in SM are often anonymous, unfiltered, and uninfluenced [19] and may offer insights from other key stakeholders, such as caregivers. These types of data are not frequently available in the published literature. Social media listening (SML) has emerged as a valuable tool that uses technology to automatically monitor, track, review, and analyze conversations and interactions taking place on different SM platforms. Such a methodology has the capability to identify patients’ unmet needs and helps better understand their lived experiences with the disease. SML has been used in recent years across several conditions, including chronic obstructive pulmonary disease [20], presbyopia [21], Parkinson disease [22], bronchiectasis [23], inflammatory bowel disease [24], COVID-19 [25], and cancer [19,26-28]. These studies highlighted the value of SML in gathering and analyzing large volumes of real-world stakeholder-centered data that are available on SM channels. Such analyses have helped uncover the most troublesome disease symptoms, considerations behind patients’ choice of available treatment options, the impact of disease and treatment on QoL and emotional well-being, and financial repercussions associated with disease burden, among other factors. To our knowledge, there is no published literature on the use of SML to understand the lived experiences and needs of patients with GVHD. This study aimed to explore how GVHD stakeholders, including patients, caregivers, and health care professionals (HCPs), describe their experiences using SM. Furthermore, it explored the needs and perceptions using SML analysis to generate patient insights from across 14 European countries in terms of treatments received, predictors of outcome, treatment effectiveness and safety, and burden of illness.

## Methods

### Data Collection and Search Strategy

This study was a retrospective analysis of SM data freely available in the public domain. Data around GVHD-specific terms were collected retrospectively for 24 months from April 2019 to April 2021 across 14 European countries (the United Kingdom, Spain, France, Switzerland, Belgium, Germany, Austria, the Netherlands, Italy, Nordic countries [Denmark, Finland, Norway, and Sweden], and Portugal), in the following languages: English, Spanish, French, German, Dutch, Italian, Portuguese, Swedish, Norwegian, Danish, and Finnish. Predefined search strings were developed in each language to identify GVHD posts and conversations, including Boolean operators (AND, OR) to combine keywords within the search strings (Table S1 in Multimedia Appendix 1). The search string terms were originally identified through a literature review into the GVHD therapy area and a review of 2 web-based forums, Onmeda [29] and HealthUnlocked [30], which are the most frequently used health portals for sharing patients’ and caregivers’ experiences across European countries.

The SM aggregator tool, Talkwalker social analytics database [31], was used to collect data from SM posts for all included markets using the predefined search terms. A list of keywords was created to help identify and collect conversations on the topic of interest. These keywords were then used to create search strings that eventually formed a comprehensive search query, which was entered into the SM aggregator tool to streamline the search. Key information collected included demographics and any information on predefined research categories relating to the patient journey (Table S2 in Multimedia Appendix 1). Hashtags included within the search strings (Table S1 in Multimedia Appendix 1) were identified by the aggregator tool. All SM sources were included in the aggregator tool at setup. SM sources based on retrieval of information were open-access forums and blogs and social networking sites, including Twitter, Facebook (public), Instagram (public), and YouTube. SM data collected from all publicly available SM sources were evaluated for relevance to the topic of GVHD using the aggregator tool, and those open-access forums and blogs that provided the most relevant conversations were included in the study (Table S3 in Multimedia Appendix 1). Relevant posts were downloaded and tagged by channel and GVHD stakeholder, including patients, caregivers, and HCPs, and other stakeholders were also noted. Posts relating to specific stakeholders were identified based on the following predefined criteria: (1) SM users who mentioned that they are patients or have been diagnosed with GVHD and are looking for advice were defined as patients; (2) users who mentioned that their loved ones are affected with the disease and they are seeking disease-related information on behalf of their loved ones were defined as caregivers; and (3) HCPs were those users who identified themselves as doctors treating a patient or patients with GVHD; in Twitter posts, an HCP was identified using a publicly available bio associated with the Twitter profile (HCP/specialist). Posts that were originally written in languages other than English were analyzed and translated by local language specialists.

### Data Analysis

A 3-tier technique was used to identify relevant data (Figure S1 in Multimedia Appendix 1) for the final deep-dive analysis. Using the predefined search terms (Table S1 in Multimedia Appendix 1), SM posts were identified from included countries and downloaded to form the raw data set (known as the data universe) of total posts for each geographical region from all stakeholders. Exclusion of irrelevant posts was carried out by an automated relevancy approach containing keyword-based relevancy algorithms, and manual review against predefined inclusion and exclusion criteria (Table S4 in Multimedia Appendix 1) forming the contextualized data set. Further information on Data Analysis is detailed in the Methods in Multimedia Appendix 1.

### Definitions

In this study, the following definitions were used: a stakeholder is defined as a person who plays a role in the entire disease landscape and can include patients, caregivers, HCPs, researchers, patient support groups, and others. Positive or negative sentiments were defined as positive or negative mentions regarding treatment, for example, if a treatment is discussed in a positive or negative light. Treatment discontinuation was defined as a patient’s or an HCP’s action to stop treatment due to intolerable side effects or due to disease improvement. Unmet needs were defined as gaps perceived to exist in the care system by patients and caregivers, although specific unmet needs were not predefined before the study.

### Ethical Considerations

All data utilized and presented in the present SML study were obtained from publicly accessible sources without accessing password-protected information. Nevertheless, ethical aspects of SML research should be considered, as patients affected by GVHD and other stakeholders did not formally consent to their discussions being used in data collection and analyses. In general, the privacy aspect is a major concern in SML studies. Despite the lack of clear guidance on how to deal with the lack of consent or anonymity of participants used in SML research, some recommendations have been published, stating that data should be collected only to answer specific research questions and presented in such a way that identification of a participant is minimized [32]. Publicly available posts used in this study were anonymized, and any information that could identify a GVHD stakeholder (such as usernames) was removed before analysis.

This study received internal pharmacovigilance approval [registry ID DE006979 (V1)] by Novartis AE and safety reporting team. All methods were performed in accordance with the relevant guidelines and regulations involving the secondary use of social media research.

## Results

### Overview of Analyzed Social Media Posts

The data universe extracted from the initial search using predefined keywords consisted of 9016 SM posts. Of these, 325 posts were identified as contextualized data relevant to study objectives and key research questions. Due to a low number of relevant posts containing records from key stakeholders (N=325), all posts were used for deep-dive analysis (Figure S1 in Multimedia Appendix 1). The countries contributed the following number of posts toward the contextualized data: United Kingdom, n=166; France, n=51; Germany, n=51; Spain, n=17; the Netherlands, n=11; the Nordic countries (Denmark, Finland, Norway and Sweden), n=8; Italy, n=7; Belgium, n=5; Switzerland, n=4; Portugal, n=3; and Austria, n=2. Due to the lower number of posts contributed by countries except the United Kingdom, France, and Germany, key findings from the study will be discussed generally.

**Figure 1:**
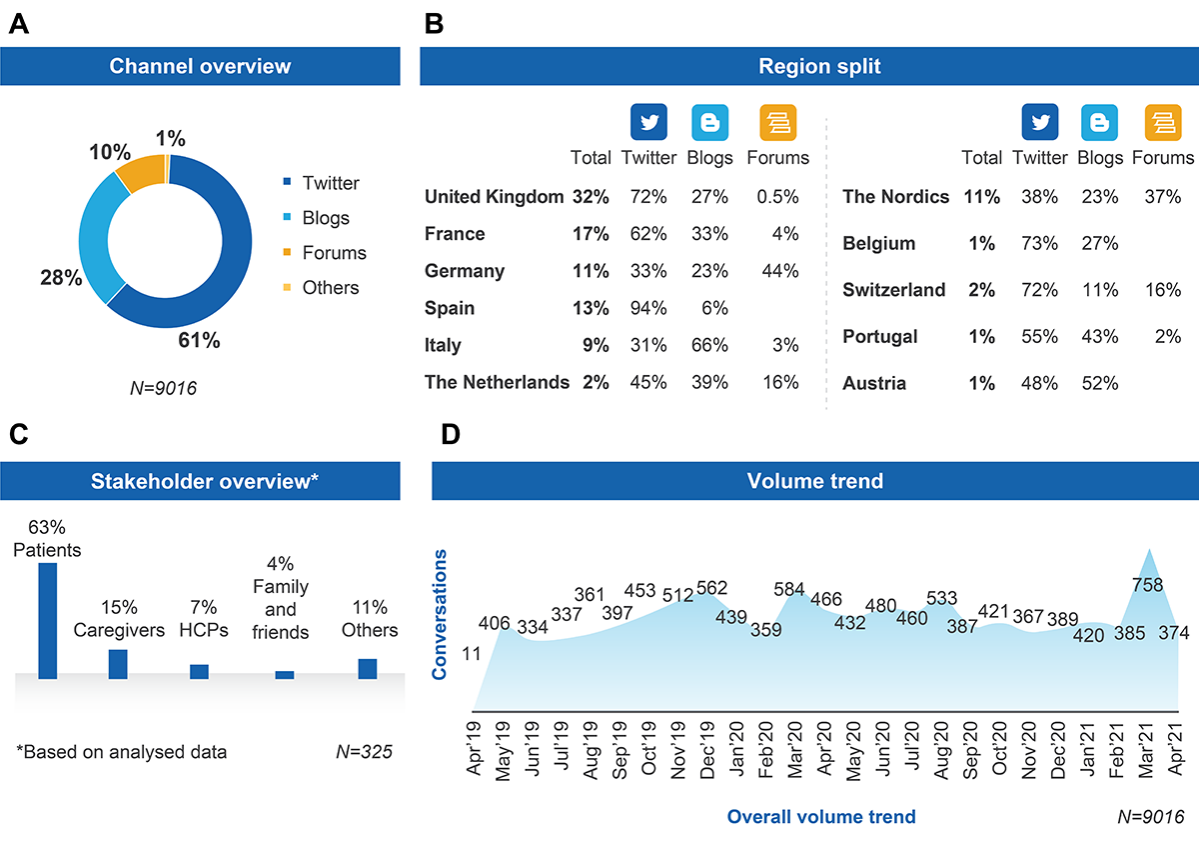
Data source and country of origin of relevant posts: (A) data source of relevant posts; (B) country of origin of relevant posts; (C) stakeholders for analyzed posts; and (D) data volume trend for relevant posts over 24 months. HCP: health care professional.

Broad search term criteria allowed us to gather posts containing any conversations mentioning specific terms for GVHD across different SM channels (N=9016). Overall, Twitter was the most popular SM channel used, contributing to most of the overall volume around GVHD (5500/9016, 61%), followed by blogs (2524/9016, 28%), and forums (902/9016, 10%; Figure 1A). The majority of these conversations were generic discussions about GVHD. Curation of this raw data set using automation and manual relevancy checks reduced the number of posts to 325, resulting in a data set rich in patients’ experiences and relevant to the research questions (Figure S1 in Multimedia Appendix 1). Of the 325 analyzed posts, Twitter contributed 250 (77%) posts; blogs, 49 (15%) posts; forums, 13 (4%) posts; Facebook, 7 (2%) posts; and Instagram and YouTube, 4 (1%) posts each. The number of posts retrieved from Facebook may have been impacted by restricted data access imposed by Facebook’s application programming interface.

Twitter was the most prominent channel for the United Kingdom (2077/2885, 72%), France (950/1533, 62%), Spain (1102/1172, 94%), the Netherlands (81/180, 45%), the Nordic countries (377/992, 38%), Belgium (66/90, 73%), Switzerland (130/180, 72%), and Portugal (50/90, 55%). Blogs were the most prominent channel used for discussions in Italy (536/811, 66%) and Austria (47/90, 52%), whereas forums were the most prominent channel used in Germany (436/992, 44%; Figure 1B). The main contributor to the overall extracted data was the United Kingdom (2885/9016, 32%), followed by France (1533/9016, 17%), Spain (1172/9016, 13%), Germany and the Nordic countries (992/9016, 11% each), and Italy (811/9016, 9%). Fewer SM posts originated from the Netherlands and Switzerland (180/9016, 2% each), and Belgium, Portugal, and Austria (all 90/9016, 1%; Figure 1B).

From the analyzed data (N=325), patients with GVHD were the primary stakeholders across Europe, contributing 63% (205/325) of SM posts (Figure 1C). The second most prominent stakeholder group discussing GVHD was caregivers (49/325, 15%), followed by HCPs (23/325, 7%), and friends and family (13/325, 4%). Other stakeholders were categorized as miscellaneous and included organizations, communities, patient support groups, and experts, all of which were responsible for 11% (35/325) of posts. For overall extracted data, peaks in SM discussions were observed in March 2020 (584 posts) and March 2021 (758 posts; Figure 1D).

**Figure 2:**
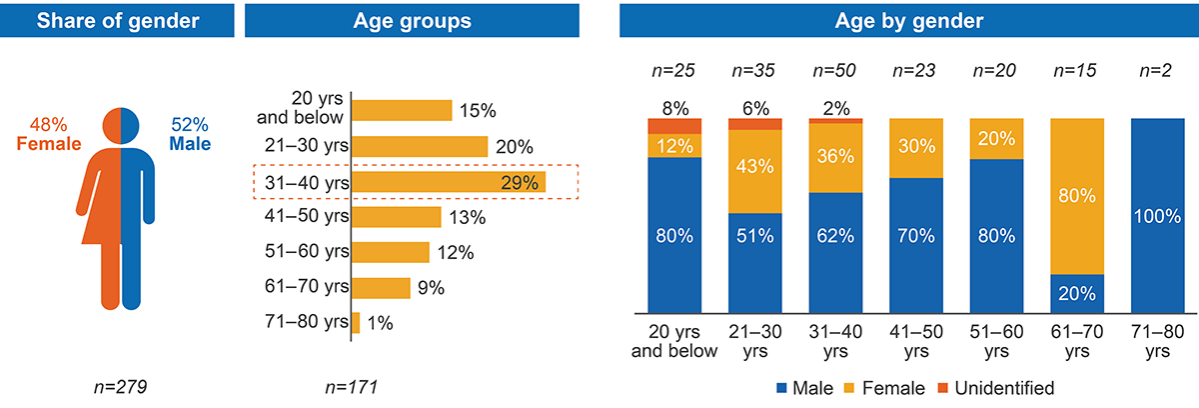
Age and gender of contributors in relevant posts. yrs: years.

Gender was identifiable in 86% (279/325) of analyzed posts, with male contributors being slightly more prominent (145/279, 52%) than female contributors (134/279, 48%). Age was identifiable in 53% (171/325) of analyzed posts, with 31-40 years identified as the most common age range (Figure 2). Demographics of the SM population from analyzed posts (N=325) are shown in Figure S2 in Multimedia Appendix 1.

### The Patient Journey in GVHD

This study provided key insights into the patient journey of those living with GVHD. Within the GVHD patient journey across Europe, analysis of 234 posts revealed that treatment was the most discussed stage (159/234, 68%), followed by symptoms (77/234, 33%), and diagnosis and tests (49/234, 21%; Figure S3 in Multimedia Appendix 1).

### Treatment

**Figure 3:**
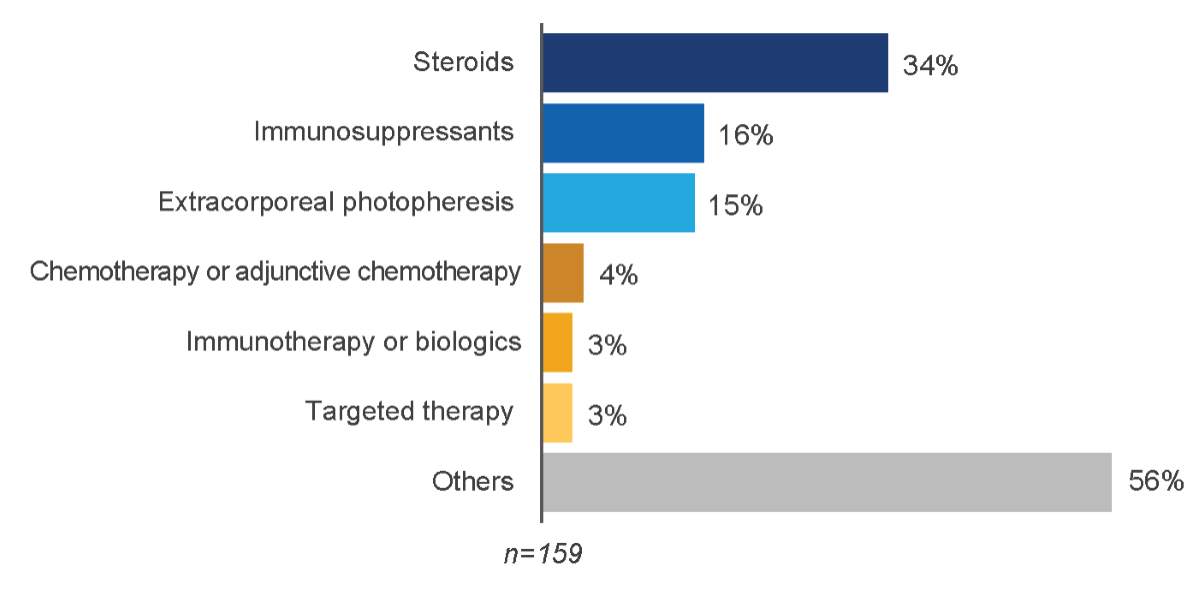
Treatments mentioned in relevant posts.

For all 14 countries included in the analysis, discussion of treatment was evident in ≥50% (159/325) of the analyzed posts. Of treatment-related posts (n=159), steroids were the most common treatment for all countries (54/159, 34%), and conversations related to steroids were commonly associated with patients younger than 60 years of age. Immunosuppressants were the second most common treatment mentioned (25/159, 16%), followed closely by extracorporeal photopheresis (24/159, 15%; Figure 3). Other key treatment types (89/159, 56%) included generic mentions of treatment, medications, drugs in general, and alternative measures. Country-specific mentions of treatments are shown in Figure S4 in Multimedia Appendix 1*.* Alternative measures (5/159, 3%) of treatment included cannabis oil, curcuma supplements, and vitamins for the management of specific GVHD types, including cGVHD, eye-related GVHD, and steroid-resistant GVHD, respectively.

### Treatment Sentiment

Stakeholders generally mentioned treatment options in a neutral tone, without positive or negative sentiment (Table 1). Of the 54 posts discussing steroids, side effects associated with these resulted in a relatively high negative sentiment (22/54, 41%; vs 7/54, 13% positive and 25/54, 46% neutral). Insights suggested that patients found it inconvenient to take additional medications to manage side effects. Efficacy led to positive sentiment in 13% (7/54) of posts, especially for skin GVHD. Similarly, for immunosuppressants, efficacy drove positive sentiments, while side effects drove negativity. Negativity around extracorporeal photopheresis was comparatively low (2/24, 8%), with patients experiencing relatively few or manageable side effects, such as looking tired for a few days and being more sensitive to the sun.

**Table 1:** Treatment features mentioned in relevant posts.

Treatment types	Positive sentiment, n (%)	Negative sentiment, n (%)	Neutral sentiment, n (%)	Total, n
Steroids	7 (13)^a^	22 (41)^b^	25 (46)^c^	54
Immunosuppressants	3 (12)^a^	5 (19)^b^	18 (69)^c^	26
Extracorporeal photopheresis	5 (21)^b^	2 (8)^a^	17 (71)^c^	24
Chemotherapy or adjunctive chemotherapy	1 (14)^b^	1 (14)^b^	5 (71)^c^	7
Immunotherapy or biologics	1 (20)^b^	2 (40)^c^	2 (40)^c^	5
Targeted therapy	1 (25)^b^	1 (25)^b^	2 (50)^c^	4

^a^Low prevalence.

^b^Medium prevalence.

^c^High prevalence.

### Treatment Features

**Figure 4:**
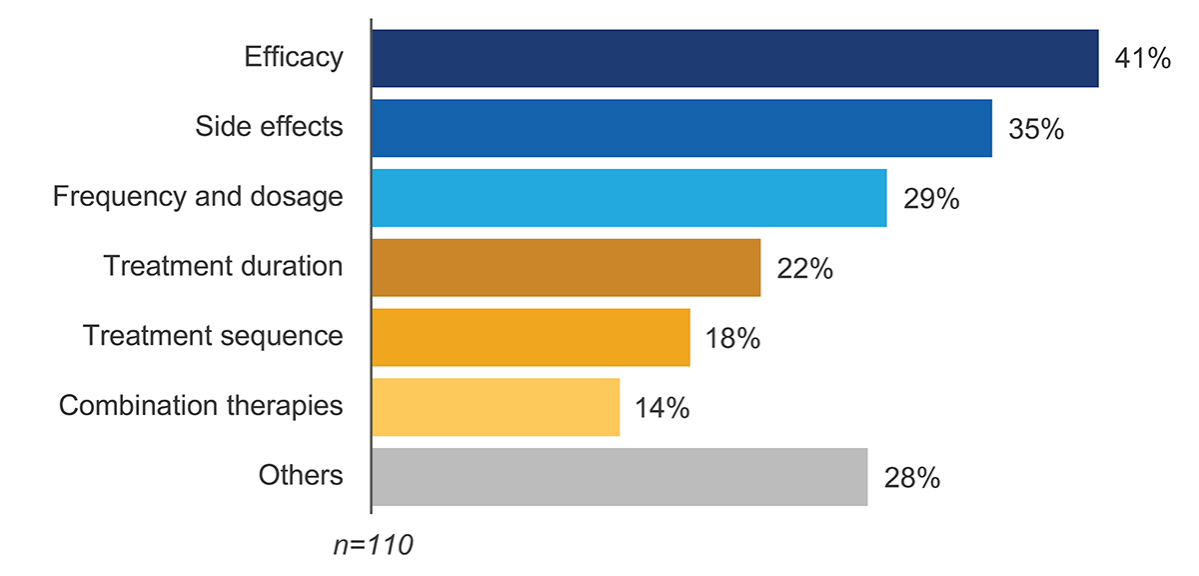
Treatment topics.

Efficacy, side effects, and frequency and dosage were the most frequently addressed treatment topics across 110 posts (45/110, 41%; 39/110, 35%; and 32/110, 29%, respectively; Figure 4). Country-specific mentions are detailed in Figure S5 in Multimedia Appendix 1.

Duration of treatment was mentioned in 7% (24/325) of analyzed conversations and 15% (24/159) of treatment-related conversations. Around 29% (7/24) of patients were on treatment for less than 30 days, which was most commonly associated with steroids. About 21% (5/24) of patients had been on treatment for their GVHD for more than 1 year, with 8% (2/24) over 5 years. A total of 95% (19/20) of posts were classified as discussions on first-line therapy, 55% (11/20) on second-line therapy, and 10% (2/20) on third-line therapy; there were no posts on fourth-line treatment. Steroids and immunosuppressants were mostly used as first-line treatments across countries, although these were also used as second-line treatments with biologics in some cases. Discussion of treatment discontinuation was rare in GVHD, with mentions in only 1% (3/325) of analyzed conversations.

### Clinical End Points

Clinical end points were mentioned in 31 (10%) of the 325 analyzed posts. Patients mostly mentioned feeling better or their condition improving in general terms as their end goals. Other end points identified in the analyzed posts included prolonged survival, reduced symptoms, improved QoL, remission, and mortality.

### Symptoms

Patients described symptoms associated with their GVHD in 77 (24%) of the 325 analyzed posts. Symptoms reported from Europe included skin-related conditions such as rash, redness, itchiness, discoloration, and dryness (49/77, 64%), dry eyes or vision change (13/77, 17%), pain and cramps (16/77, 21%), and fatigue or muscle weakness (12/77, 16%).

### Quality of Life

**Figure 5:**
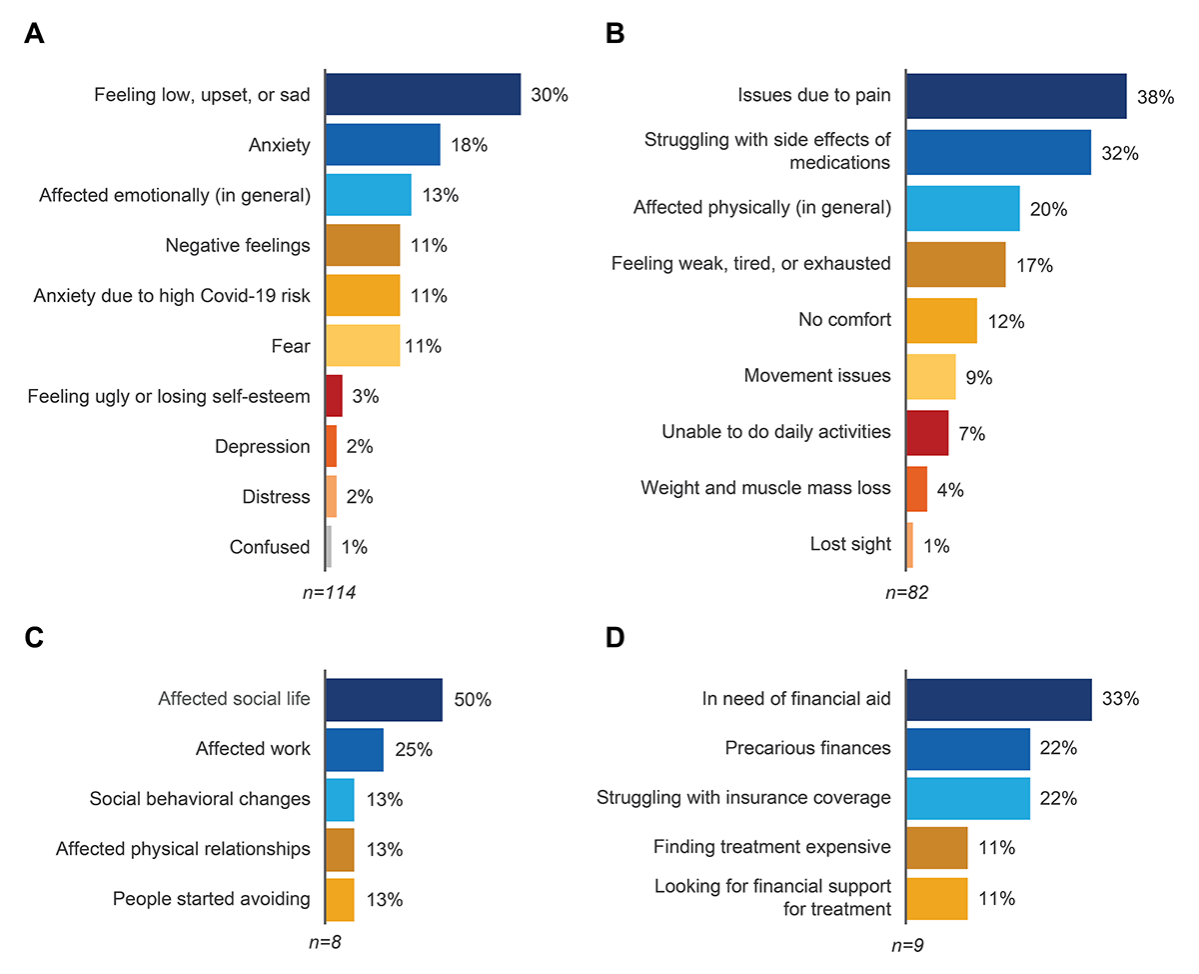
Impacts of graft-versus-host disease (GVHD) reported on social media: (A) emotional impact; (B) physical impact; (C) social impact; and (D) financial impact.

A total of 165 (51%) of the 325 analyzed posts referred to the impact of GVHD on QoL. Of these posts, the following impacts were discussed: emotional (114/165, 69%), physical and functional impact (82/165, 50%), social (8/165, 5%), and financial (3/165, 2%). Feeling low, sad, or upset (34/114, 30%), anxiety (21/114, 18%), feeling emotionally affected (15/114, 13%), and negative feelings, such as anxiety due to COVID-19 and fear (13/114, 11% all), were the most frequently reported emotional impacts (Figure 5A). Pain (31/82, 38%), struggles with side effects of medications (26/82, 32%), being physically affected (16/82, 20%), feeling weak, tired, or exhausted (14/82, 17%), and having no comfort (10/82, 12%) were the most frequently reported physical impacts (Figure 5B). Affected social life (4/8, 50%) and affected work life (25%, 2/8) were the most frequently reported social impacts (Figure 5C). Needing financial aid (3/9, 33%), precarious finances, and struggling with insurance coverage (2/9, 22% each) were the most frequently reported financial impacts (Figure 5D).

### Unmet Needs

**Figure 6:**
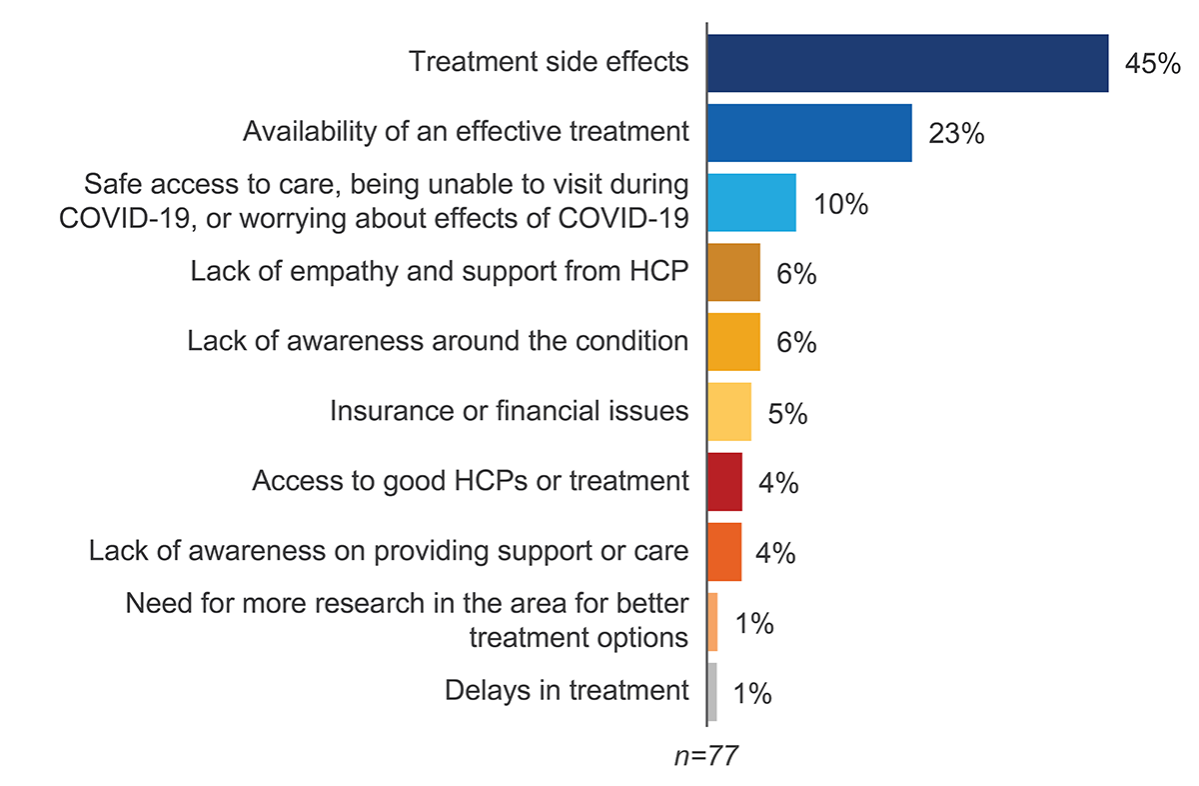
Key unmet needs of graft-versus-host disease (GVHD) stakeholders in Europe. HCP: health care professional.

Unmet needs were mentioned by patients or caregivers in 24% (77/325) of the analyzed conversations. Treatment side effects (35/77, 45%), availability of an effective treatment (18/77, 23%), and safe access to care during the COVID-19 pandemic (8/77, 10%) emerged as key unmet needs of patients with GVHD and other stakeholders (Figure 6). Reported side effects ranged from mild (eg, sleeplessness and weight gain) to severe, including steroid-induced diabetes, loss of large bowel function, and weakness.

Other unmet needs included a lack of empathy and support from HCPs, a lack of awareness around GVHD, financial concerns, access to good HCPs or treatment, a lack of awareness about providing support and care, the need for research into better treatment options, and delays in treatment (Figure 6).

## Discussion

### Overview

To our knowledge, this study provides the first qualitative insights into how the journey of a patient with GVHD is discussed on the web by multiple stakeholders and identifies key concepts relevant to individuals living with GVHD across Europe. European countries with larger populations (the United Kingdom, France, and Germany) were the highest contributors to the overall and relevant posts included in the study. This could suggest that the number of patients with GVHD in these countries is proportionally higher than in countries included in the study that have smaller populations, but it could also suggest varied usage of SM across countries. Furthermore, the number of stem cell transplantations across Europe continues to rise [33], suggesting that the prevalence of GVHD could continue to rise and SM usage may increase.

The total number of relevant posts (N=325) identified in SML was limited. However, there are several possible explanations for this, including high levels of distress and burnout, which can be experienced by patients and their parents, particularly in a pediatric setting [34,35]. Such feelings may prevent patients or caregivers from wanting to discuss their GVHD further. Furthermore, older adults and very young recipients may not use SM tools, suggesting these patient populations may be underrepresented in this study.

After a deep-dive analysis of 325 relevant posts, treatment was the most discussed stage, followed by symptoms and diagnosis within the patient journey. Steroids were the most reported therapy, as expected in line with published literature [5], and some negativity surrounding their use is unsurprising given the safety profile of these medications. Side effects from steroids are widely acknowledged [36], particularly at higher doses and with a longer duration of therapy [37], highlighting the need for improved supportive care [38] and multidisciplinary management [39,40], particularly for those with cGVHD. Future novel therapies and approaches for GVHD may see a shift away from steroid therapy, reducing the possibility of unwanted side effects [38,41].

This study identified emotional impact of the disease as a frequently discussed topic within the analyzed posts (165/325, 69%) across patients with GVHD, followed by a high physical impact across patients with GVHD within the analyzed posts. This finding may support the notion that patients often turn to SM for community support and advice in times of distress or lowered mood. It is documented that impaired QoL and functional status occur across GVHD [11-14], in particular the high emotional impact, in which feeling low, sad, or upset is highlighted in several QoL studies [15,16]. Together, these findings demonstrate the need to further understand the negative emotional impact of GVHD, how QoL can be improved, and what support can be provided for this patient population. The availability of web-based tools and programs for patients with GVHD may offer opportunities to improve outcomes, including mood, as demonstrated by the “INternet-based Survivorship Program with Information and REsources” (INSPIRE) for survivors of HSCT [42]. It is also important to recognize that among all symptoms (including skin-related conditions) most discussed by stakeholders within this study, pain and fatigue had the main physical impact on QoL.

The SM data analyzed in this study were collected from both the prepandemic and the COVID-19 pandemic periods. The multiple effects of the COVID-19 pandemic on patients’ well-being and their lived experiences may have impacted the results of this study. The lack of safe access to HCPs and, in most cases, face-to-face consultations with HCPs, being quarantined, and being worried about the health implications of COVID-19 may have heightened stakeholders’ sensitivity and impacted their emotional well-being. Indeed, this study identified safe access to care during the COVID-19 pandemic as one of the key unmet needs of patients with GVHD and other stakeholders. However, this study did not perform stratification and analysis of SM posts in the prepandemic and pandemic periods, and further research is needed to address whether there were significant differences in stakeholders’ unmet needs and patients’ symptom burden during the year leading to the outbreak of the COVID-19 pandemic and during the pandemic.

In this study, the key topics of SM discussions were received treatments, various treatment features (efficacy, side effects, frequency, and dosage), disease symptoms, QoL, and unmet needs. This type of data can provide a rich knowledge landscape and complement the data collected using more conventional survey approaches. Web-based data collection systems provide valid means to investigate different aspects of GVHD but often address issues surrounding only clinical aspects of the disease, for example, diagnostic precision and certainty, and are mostly aimed at HCPs [43,44]. Questionnaire-based surveys and interviews may not be the most effective methodology for gathering large amounts of data in a time-effective manner, and study outcomes are usually based on a small patient population [45,46]. Moreover, the restrictive nature of such surveys in terms of the breadth of topics is a drawback. In contrast, SML can be easily tailored to study objectives of interest, capturing either largely unfiltered stakeholder-related data or being tuned to answer specific research questions. This study demonstrated that SML can identify important topics relating to both clinical and QoL aspects of living with GVHD that may not be available in published studies using more conventional data collection and analysis methodologies. It is also noteworthy that patients with rare medical conditions, such as GVHD, may find SM particularly accommodating for sharing their disease-associated experiences, especially when patient populations are geographically distant [47].

Using SM may help improve patient-physician interactions, encourage informed and shared decision-making, improve treatment options by further understanding unmet needs, and increase patient satisfaction. Finally, SML may eventually assist clinical trial design by adjusting patient-reported outcome measures to better assess the impact of new therapeutic agents on improving the QoL of patients living with GVHD.

### Limitations

This study has limitations that should be considered. SM research generally assumes that the information provided by patients is authentic. The quality of insights gathered from the analysis of digital conversations is dependent on the richness of patient conversations. The SM population is not representative of the whole community affected by GVHD, with a low number of relevant conversation volumes (N=325) and the median age of posts appearing to be slightly lower than the median age of typical patients in this setting [48]. The age of SM users may be skewed toward younger than average patients, and pediatric and elderly groups may be underrepresented.

Due to limited references to technical terms, results were provided overall and not separated by disease classification, severity, or affected tissue; this may influence the interpretation of treatment patterns, QoL, and unmet needs.

Public posting might introduce bias, as people are unlikely to share very personal information through such channels. In this study, only discussions publicly available through SM platforms were used; therefore, some discussions are likely to have been missed in closed channels, which are often active. Furthermore, verbal data that could be collected from platforms such as YouTube were not used in the analysis. All data were retrospectively collected from SM posts in the public domain. In some instances, information about the SM population could not always be identified, including demographic and clinical information.

### Conclusions

This SML study further confirms that GVHD has a significant impact on patients’ daily lives. Stakeholders experience a significant emotional and physical impact that affects their QoL. Although some limitations are apparent with SML, this study provides valuable insights into the GVHD experience, complementing published evidence from traditional studies. Future SML studies should be performed using the same approach described in this study to monitor whether GVHD stakeholders express novel concerns with respect to their disease and its treatment and how stakeholders’ views and patients’ lived experiences evolve over time, particularly with regulatory approvals of novel nonsteroid therapies for GVHD. Importantly, further SML studies should strive to validate the quality of SM data with regard to GVHD diagnosis, treatment, and side effects of current therapies by evaluating the SML data against evidence-based clinical and laboratory databases. Further real-world insights will strengthen our understanding of the lived experiences of those with GVHD and may reveal unmet medical needs for this patient population.

## Appendix

Multimedia Appendix 1 Social Media Listening_Manuscript_Multimedia Appendix 1.

## Abbreviations

aGVHD: acute graft-versus-host disease
cGVHD: chronic graft-versus-host disease
GVHD: graft-versus-host disease
HCP: health care professional
HSCT: hematopoietic stem cell transplantation
INSPIRE: INternet-based Survivorship Program with Information and REsources
JAK: Janus kinase
QoL: quality of life
SM: social media
SML: social media listening

## Data Availability

All data generated or analyzed during this study are included in this published article and its Multimedia Appendix 1.
