# Supplementary material for: Understanding the Needs and Lived Experiences of Patients With Graft-Versus-Host Disease: Real-World European Public Social Media Listening Study
Source: JMIR Cancer. 2023 Nov 10;9:e42905. doi: 10.2196/42905 (PMC10674148; doi:10.2196/42905)
Supplement: Multimedia Appendix 1 [file cancer_v9i1e42905_app1.docx]

**Lived Experiences of Patients with Graft-Versus-Host Disease:**

**Real-World Insights from a European Public Social Media Listening Study**

**MULTIMEDIA APPENDIX 1**

Zinaida Perić,^1^ Grzegorz W. Basak,^2^ Christian Koenecke,^3^ Ivan Moiseev,^4^ Jyoti Chauhan,^5^ Sathyaraj Aasaithambi,^5^ Alexandros Sagkriotis,^6*^ Sibel Gunes,^6^ Olaf Penack^7^

**Affiliations:**

^1^School of Medicine, University of Zagreb and University Hospital Center Zagreb, Zagreb, Croatia

^2^Medical University of Warsaw, Warsaw, Poland

^3^Hannover Medical School, Hannover, Germany

^4^RM Gorbacheva Research Institute, Pavlov University, St. Petersburg, Russian Federation

^5^Novartis Healthcare Pvt. Ltd, Hyderabad, India

^6^Novartis Pharmaceuticals AG, Basel, Switzerland

^7^Charité – Universitätsmedizin Berlin, corporate member of Freie Universität Berlin and Humboldt-Universität zu Berlin, Department of Hematology, Oncology and Tumor Immunology, Berlin, Germany

*Novo Nordisk Health Care AG, Zurich, Switzerland

**Corresponding author:** Professor Olaf Penack

**Email:** [olaf.penack@charite.de](mailto:olaf.penack@charite.de)

# **Multimedia Appendix Methods**

Relevancy checks ensured insights were provided in response to key research questions (Multimedia Appendix 2). Included posts were those considered to be ‘patient-centric’, including those posts by patients themselves or caregivers, or patient stories by other. Patient-centric posts were then coded based on relevance to at least one pre-defined key category (Multimedia Appendix 2).

A deep-dive analysis was performed manually on the contextualized dataset to identify insights and themes related to stakeholder perceptions on key domains: disease burden (including quality of life), epidemiology and patient characteristics, real-world effectiveness (including treatment choice and patient-reported outcomes), treatment patterns and compliance (including treatment sequence and discontinuation) and real-word safety (including treatment sentiment and side effects). Only publicly available information on mentioned sources was accessed and used for this research. All unique identifiers were anonymized/removed prior to data analysis to allow for confidentiality.

# **Table S1.** Social media search strings

| **Language** | **Search Terms Used** |
| --- | --- |
| **Dutch** | ("graft-versus-host-ziekte" OR "transplantaat versus gastheerziekte" OR "GvHD" OR "Graft versus host disease" OR "aGvHD" OR "Acute graft versus host disease" OR "cGvHD" OR "Chronic graft versus host disease" OR "Graft Host" OR (Graft NEAR/4 "host disease") OR "#gvhd" OR "#GVHD" OR "#GvHD" OR "Graft-versus-host disease" OR #Graftversushostdisease OR "Graft-versus-host-disease" OR Graftvshostdisease OR "Graft vs. host disease" OR "graft vs host disease" OR "runt disease" OR "graft-verses-host-ziekte" OR "Graft verses host disease" OR "Graft-verses-host disease" OR #Graftverseshostdisease OR "Graft-verses-host-disease") |
| **English** | ("GvHD" OR "Graft versus host disease" OR "aGvHD" OR "Acute graft versus host disease" OR "cGvHD" OR "Chronic graft versus host disease" OR "Graft Host" OR (Graft NEAR/4 "host disease") OR "#gvhd" OR "#GVHD" OR "#GvHD" OR "Graft-versus-host disease" OR #Graftversushostdisease OR "Graft-versus-host-disease" OR Graftvshostdisease OR "Graft vs. host disease" OR "graft vs host disease" OR "runt disease" OR "graft-verses-host disease" OR "graft verses host disease") |
| **French** | ("GvHD" OR "RGCH" OR "GVH" OR "MGCH" OR "maladie du greffoncontrel'hôte" OR "réaction du greffoncontrel'hôte" OR "réaction de greffecontrehôte" OR "maladie de rejet du greffon" OR "aGvHD" OR "GVHa" OR "maladieaiguë du greffecontrel'hôte" OR "maladie du greffecontrel'hôteaiguë" OR "réaction du greffoncontrel'hôteaigüe" OR "maladie du greffoncontrel'hôteaigüe" OR "maladie de rejet du greffonaigüe" OR "cGvHD" OR "GVHc" OR "maladiechronique du greffoncontrel'hôte" OR "maladie du greffoncontrel'hôtechronique" OR "maladie de rejet du greffonchronique" OR "GVH chronique" OR "GVHD chronique" OR (greffe NEAR/4 "contrel'hôte") OR (greffon NEAR/4 "contrel'hôte") OR "#gvhd" OR "#GVHD" OR "#GvHD" OR "#RGCH" OR "#GVH" OR "#MGCH" OR "#maladiedugreffoncontrel’hôte" OR "Graft Host" OR (Graft NEAR/4 "host disease") OR "#gvhd" OR "#GVHD" OR "#GvHD" OR "Graft-versus-host disease" OR #Graftversushostdisease OR "Graft-versus-host-disease" OR Graftvshostdisease OR "Graft vs. host disease" OR "graft vs host disease" OR "runt disease" OR "graft-verses-host disease" OR "graft verses host disease") |
| **German** | ("GvHD" OR "GvHR" OR “Graft versus Host disease” OR "Spender gegen Empfänger Reaktion" OR "Transplantat gegen Wirt Reaktion" OR "Transplantat gegen Wirt Krankheit" OR "Graft versus Host Reaktion" OR "aGvHD" OR "Akute Spender gegen Empfänger Reaktion" OR "Akute Spender-gegen-Empfänger-Reaktion" OR "Akute Spender-gegen-Wirt-Krankheit§" OR "chronische Spender-gegen-Wirt-Krankheit" OR "chronische Spender-gegen-Empfänger-Reaktion" OR "Akute Transplantat gegen Wirt Krankheit" OR "cGvHD" OR "Chronische Transplantat gegen Wirt Krankheit" OR "Chronische Spender gegen Empfänger Reaktion" OR "Chronische Spender gegen Empfänger Krankheit" OR "chronische GvHD" OR "akute GvHD" OR "Transplantat Wirt" OR (Transplantat NEAR/4 "Wirt Krankheit") OR "#gvhd" OR "#gvhr" OR "#GVHD" OR "#GvHD" OR "Transplantat-gegen-Wirt-Krankheit" OR "#TransplantatgegenWirtKrankheit" OR "Spender-gegen-Empfänger-Krankheit" OR "Graft-versus-host Erkrankung" OR "Graft-versus-host-Krankheit" OR "Spender-gegen-Wirt-Krankheit" OR "Spender-gegen-Wirt-Reaktion" OR "Spender-gegen-Wirt-Erkrankung" OR "Spender gegen Wirt Reaktion" OR "Spender gegen Wirt Krankheit" OR "Graft versus Host disease" OR "Spender-gegen-Empfänger-Reaktion" OR "Graft versus Host Reaktion") |
| **Italian** | ("GvHD" OR "GVHD" OR "Graft versus host disease" OR "malattia da reazioneimmunologica del trapiantocontrol'ospite" OR "aGvHD" OR "graft versus host disease acuta" OR "malattia acuta del trapiantocontrol'ospite" OR "malattiacronica del trapiantocontrol'ospite" OR "malattia acuta da reazione del trapianto verso l'ospite" OR "malattia cronica da reazione del trapianto verso l'ospite" OR "reazione del trapianto verso l'ospite" OR "malattia da trapianto control'ospite" OR "cGvHD" OR "graft versus host disease cronica" OR "Graft Host" OR (Graft NEAR/4 "host disease") OR "#gvhd" OR "#GVHD" OR "#GvHD" OR "Graft-versus-host disease acuta" OR "Graft-versus-host disease cronica" OR #Graftversushostdisease OR "Graft-versus-host-disease" OR Graftvshostdisease OR "Graft vs. host disease" OR "graft vs host disease" OR "Graft verses host disease" OR "Graft-verses-host disease acuta" OR #Graftverseshostdisease OR "Graft-verses-host-disease") |
| **The Nordics (Norwegian, Swedish, Danish and Finnish Combined)** | ("GvH" OR "Graft-versus-host-sjukdom" OR "aGvHD" OR "transplantat-mot-värdsjukdom" OR "transplantat-mot-värd" OR "cGvHD" OR "Kroniskttransplantat mot värdsjukdom" OR "Graft värd" OR graftvärd OR "#gvhd" OR "#GVHD" OR "#GvHD" OR "#transplantatmotvärdsjukdom" OR "transplantat-mot-värdsjukdom" OR "graft versus verts sykdom" OR transplantatmotvärdsjukdom OR "transplantat mot värdsjukdom" OR "Graft-versus-vært sygdom" OR "transplantat-mot-vert-sykdom" OR "siirteen tai isännäntauti" OR "siirteen ja isännän välistä taut*" OR "Siirteenjaisännän taut*") |
| **Portuguese** | (DECH OR ++"DECHa" OR ++"DECHc" OR DEVH OR "enxerto contra hospedeiro" OR "enxerto contra o hospedeiro" OR enxertocontrahospedeiro* OR enxertocontraohospedeiro* OR enxertoversushospedeiro* OR doençadoenxertovshospedeiro* OR enxertovshospedeiro* OR doençadoenxertocontrahospedeiro* OR doençadoenxertocontraohospedeiro* OR "enxerto-vs-hospedeiro" OR "enxerto-contra-o-hospedeiro" OR "enxerto-contra-hospedeiro" OR "GvHD" OR "Graft versus host disease" OR "aGvHD" OR "Acute graft versus host disease" OR "cGvHD" OR "Chronic graft versus host disease" OR "Graft Host" OR (enxerto NEAR/4 (hospedeiro)) OR (Graft NEAR/4 "host disease") OR "#gvhd" OR "#GVHD" OR "#GvHD" OR "Graft-versus-host disease" OR #Graftversushostdisease OR "Graft-versus-host-disease" OR Graftvshostdisease OR "Graft vs. host disease" OR "graft vs host disease" OR "runt disease" OR "Graft verses host disease" OR "Graft-verses-host disease" OR #Graftverseshostdisease OR "Graft-verses-host-disease") |
| **Spanish** | ("enfermedad de injerto contra receptor" OR "EICR" OR "EICR cronica" OR "EICR aguda" OR "enfermedad de injerto contra huésped" OR "EICH" OR "EICH cronica" OR "EICH aguda" OR "injerto contra el huesped" OR "GvHD" OR "Graft versus host disease" OR "aGvHD" OR "Acute graft versus host disease" OR "cGvHD" OR "Chronic graft versus host disease" OR "Graft Host" OR ((Graft OR injerto) NEAR/4 ("host disease" OR "contra huesped" OR "contra el huesped" OR "contra receptor" OR "contra el receptor")) OR "#gvhd" OR "#GVHD" OR "#GvHD" OR "Graft-versus-host disease" OR #Graftversushostdisease OR #enfermedaddeinjertocontrahuesped OR #injertocontrahuesped OR #enfermedaddeinjertocontraelhuesped OR #enfermedaddeinjertocontrareceptor OR #injertocontrareceptor OR #enfermedaddeinjertocontraelreceptor OR "Graft-versus-host-disease" OR Graftvshostdisease OR "Graft vs. host disease" OR "graft vs host disease" OR "runt disease" OR "Graft verses host disease" OR "Graft-verses-host disease" OR #Graftverseshostdisease) |

# **Table S2.** Key categories of interest within this study

| - **Characteristics of patients with GVHD**   - Patient demographics   - GVHD type and grade   - Organs affected and transplant types |
| --- |
| - **Treatment**   - Key treatment types   - Treatment pattern (1L, 2L, 3L)   - Treatment discontinuation   - Duration on treatment / time on treatment   - Treatment features (effectiveness, side effects etc.)   - Steroid usage (dose, escalation/de-escalation, interruptions)   - Clinical endpoints   - Patients taking ruxolitinib |
| - Hospitalization/hospital visits |
| - Impact of GVHD on patient lives (emotional, physical/functional, social, economic) |
| - Patient journey (diagnosis to remission/end of life) |
| - Key unmet needs and challenges |

1L, first-line; 2L, second-line; 3L, third-line; GVHD, graft-versus-host disease.

# **Table S3.** Sources of forum and blog posts

| **Type of Social Network** | **Forum URL** |
| --- | --- |
| Forum | [https://www.krebs-kompass.de](https://urldefense.com/v3/__https:/www.krebs-kompass.de/__;!!N3hqHg43uw!_NYQuFBCAQIGkgoObSr3hatIpOauEIC7q4mt6cHFuzcfx0PGr5eLzM5eUdoWzUn5gWob$) |
|  | [https://www.onmeda.de/forum](https://urldefense.com/v3/__https:/www.onmeda.de/forum__;!!N3hqHg43uw!_NYQuFBCAQIGkgoObSr3hatIpOauEIC7q4mt6cHFuzcfx0PGr5eLzM5eUdoWzW45uPpK$) |
|  | [https://melius.club/topic](https://urldefense.com/v3/__https:/melius.club/topic__;!!N3hqHg43uw!_NYQuFBCAQIGkgoObSr3hatIpOauEIC7q4mt6cHFuzcfx0PGr5eLzM5eUdoWzUs0fioq$) |
|  | [https://forum.psv.nl](https://urldefense.com/v3/__https:/forum.psv.nl/__;!!N3hqHg43uw!_NYQuFBCAQIGkgoObSr3hatIpOauEIC7q4mt6cHFuzcfx0PGr5eLzM5eUdoWzb3KzslZ$) |
|  | [https://forum.hardware.fr](https://urldefense.com/v3/__https:/forum.hardware.fr/__;!!N3hqHg43uw!_NYQuFBCAQIGkgoObSr3hatIpOauEIC7q4mt6cHFuzcfx0PGr5eLzM5eUdoWzbjl9LGH$) |
|  | https://healthunlocked.com/ |
| Blog | [https://geoffbeatsleukemia.com](https://geoffbeatsleukemia.com/) |
|  | [https://lifeloveandleukemia.blog](https://lifeloveandleukemia.blog/) |
|  | [https://blog.anthonynolan.org](https://blog.anthonynolan.org/) |
|  | [http://geudertheim.blogspot.com](http://geudertheim.blogspot.com/) |
|  | [https://www.sy-marlin.de](https://www.sy-marlin.de/) |
|  | [https://donaldsyear.wordpress.com](https://donaldsyear.wordpress.com/) |
|  | <http://scienceblogs.de/nucular/> |
|  | [https://igotthis.home.blog](https://igotthis.home.blog/) |
|  | [http://merelveraa.blogspot.com](http://merelveraa.blogspot.com/) |
|  | <https://michaelheeren.home.blog/> |
|  | [https://marleenvanamerongen.blogspot.com](https://marleenvanamerongen.blogspot.com/) |
|  | [https://meindertvandijkleukemie.blogspot.com](https://meindertvandijkleukemie.blogspot.com/) |
|  | [https://consultoria-estrategica.blogspot.com](https://consultoria-estrategica.blogspot.com/) |
|  | [https://www.quenoteladen.es](https://www.quenoteladen.es/) |
|  | [https://goejsen.wordpress.com](https://goejsen.wordpress.com/) |
|  | [https://minkampmotaml.blogg.se](https://minkampmotaml.blogg.se/) |
|  | <https://pantareii.wordpress.com> |
|  | https://contosdameninamulher.blogs.sapo.pt |

#

# **Table S4.** Pre-defined inclusion and exclusion criteria

| **Inclusion criteria** | - Posts included within the final analysis:   - GVHD patient cases, either authored by themselves or by the caregivers, friends/family, HCPs, or any other stakeholders   - Contained information about GVHD, specifically; causes, symptoms experienced, diagnosis, treatment, management, disease endpoint (remission, recurrence, death), QoL impact, and unmet needs |
| --- | --- |
| **Exclusion criteria** | - Posts excluded from the final analysis:   - Not patient cases   - Only contained non-insightful content; simple mentions of GVHD without insight   - Contained buy/sell content or market reports   - Contained animal content   - Were job postings   - Were link duplicates |

GVHD, graft-versus-host disease; QoL, quality of life.

# **Figure S1.** Three-tier process for data analysis


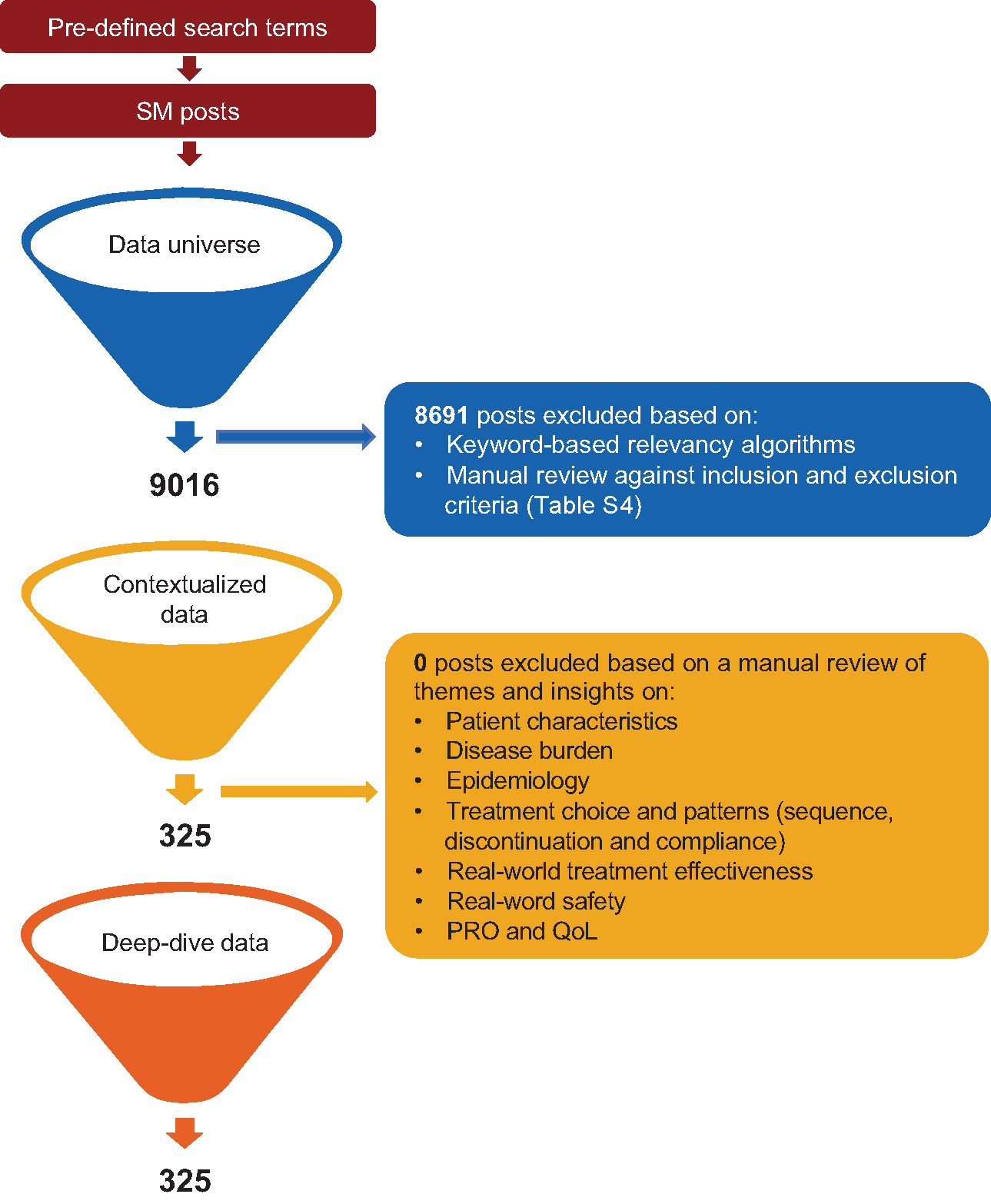


PRO, patient-reported outcomes; QoL, quality of life, SM, social media.


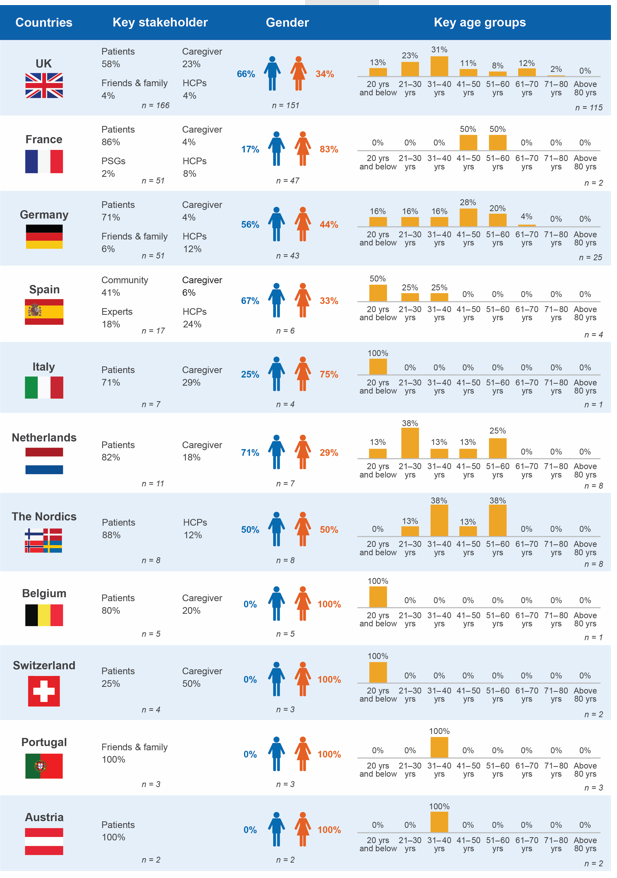
**Figure S2.** Stakeholder demographics by country (N=325)

HCPs, health care professionals; PSGs, patient support groups; yrs, years.

# **Figure S3.** Patient journey stages (n=234)


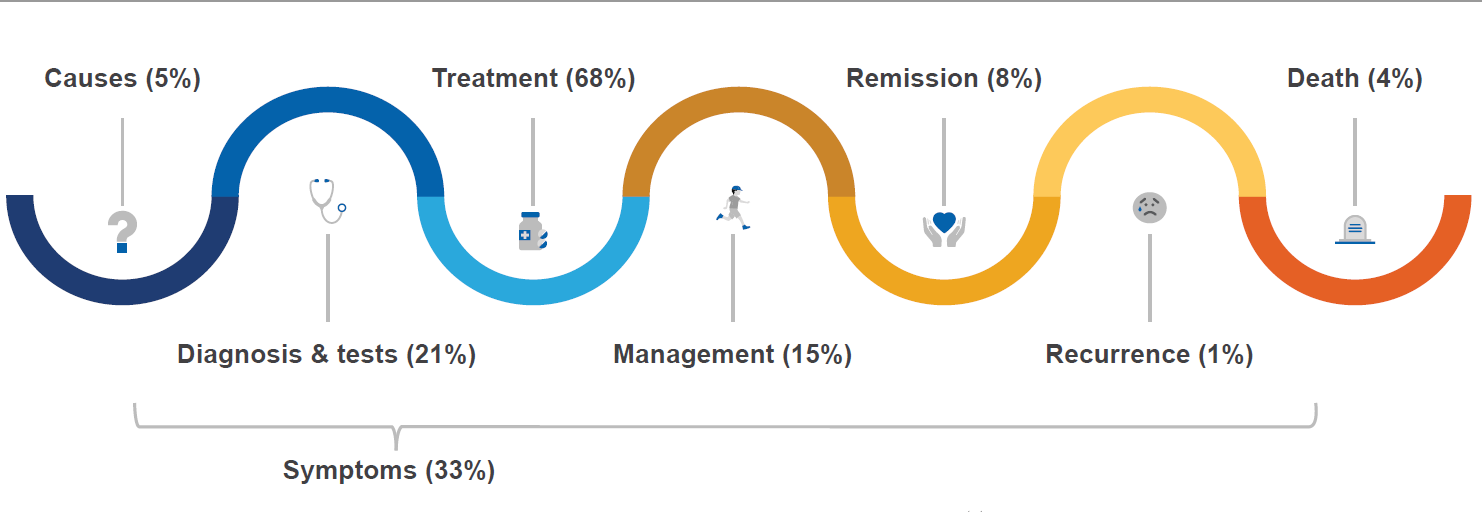


# **Figure S4.** Treatment mentions by country


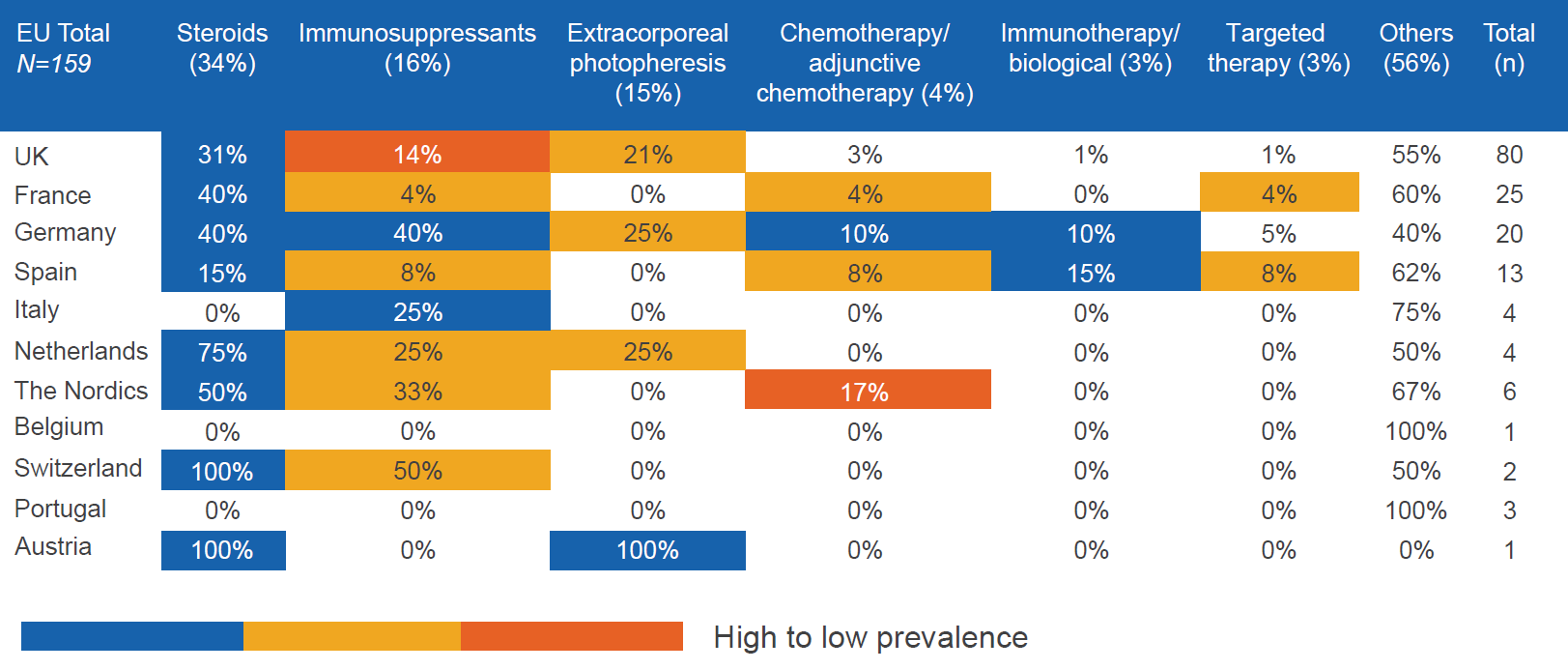


‘Others’ included generic mentions of treatment, medications, and alternative measures.

# **Figure S5.** Treatment feature topics of discussion by country


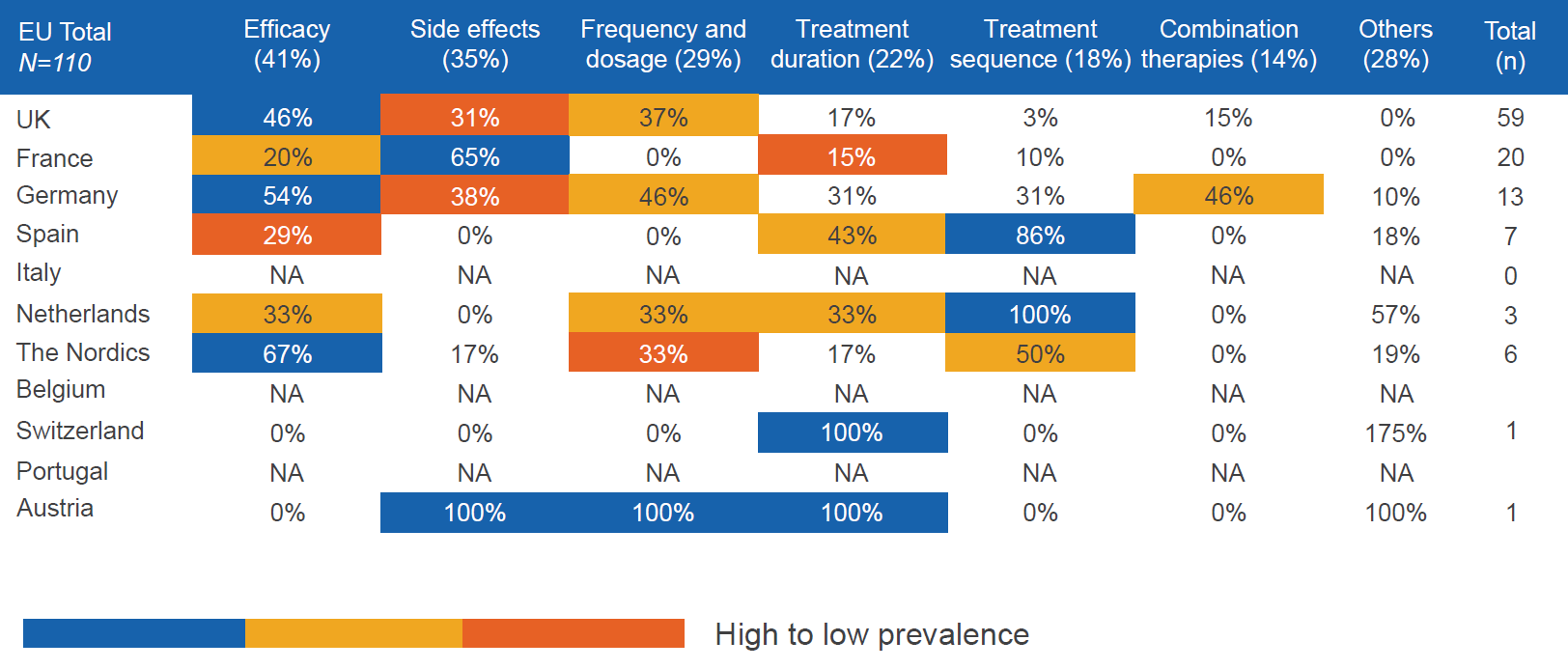


NA, not available.

‘Others’ included inefficacy, availability and access, treatment discontinuation, cost, treatment postponed/rescheduled and medicine resistance.
